# Telemedicine: an Effective and Low-Cost Lesson From the COVID-19 Pandemic for the Management of Heart Failure Patients

**DOI:** 10.1007/s11897-023-00624-y

**Published:** 2023-09-04

**Authors:** Paolo Severino, Silvia Prosperi, Andrea D’Amato, Claudia Cestiè, Vincenzo Myftari, Viviana Maestrini, Lucia Ilaria Birtolo, Domenico Filomena, Marco Valerio Mariani, Carlo Lavalle, Roberto Badagliacca, Massimo Mancone, Francesco Fedele, Carmine Dario Vizza

**Affiliations:** Department of Clinical, Internal, Anesthesiology and Cardiovascular Sciences, Sapienza University of Rome, Viale del Policlinico, 155, 00161 Rome, Italy

**Keywords:** Telemedicine, Heart failure, Prevention, Guideline-directed medical therapy, Prognosis

## Abstract

**Purpose:**

The purpose of this review is to explore the benefits and controversies that telemedicine (TM), applied to patients with heart failure (HF), can provide in terms of diagnosis, therapeutic management, and prognosis improvement.

**Recent Findings and Summary:**

During the coronavirus disease 19 (COVID-19) outbreak, TM emerged as the most effective and feasible method available to ensure continuous care for chronic diseases. Among these, HF, characterized by high mortality, morbidity, and the need for frequent visits, may benefit of the TM role. HF patients are affected by frequent exacerbations undergoing a progressive prognosis impoverishment, strongly depending on the disease’s management. A precise clinical handling is always required, with a constant optimization of the therapy, a continuous control of risk factors, and a sensitive attention to any change in symptoms, clinical signs, and laboratory tests. In this context, TM has shown to improve therapy adherence and HF: patients’ self-care, impacting the prognosis even if specific results are controversial. Major evidence shows that TM may allow an adequate primary prevention, reducing the impact of the main cardiovascular risk factors. TM can also be useful for the secondary prevention, early detecting a likely HF exacerbation before it becomes clinically manifest, thereby lowering the need for hospitalization. Moreover, an optimal up-titration of the therapy and an increase in treatment adherence are feasible by using TM. However, some studies did not show unambiguous results, and uncertainties still remain.

**Graphical Abstract:**

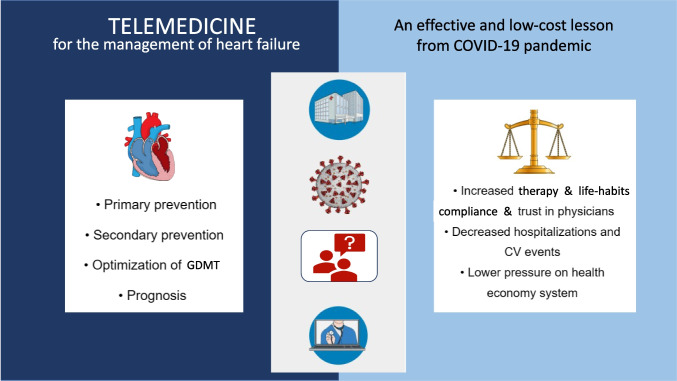

## Introduction

Telemedicine (TM), defined as the use of technology to connect patients to healthcare professionals, has been developed since 1970s. Over the past 50 years, the ways in which TM has been applied have enormously evolved, as well as its goals. Originally, the aim of TM was to provide adequate healthcare to remote populations [1]; nowadays, instead, it seeks to a proper cost-effectiveness ratio, maintaining high quality of care, reducing patient travel costs, and minimizing the frequency of clinic visits still facilitating rapid access to care [2].

The benefits that TM seemed to have for chronic disease management were already known [3], and, since then, the number of papers about this topic has significantly increased [4]. The turning point then occurred with the coronavirus disease 19 (COVID-19) outbreak and the related lockdown, which had a great impact on heart failure (HF) patients from both a medical and psychological point of view. In this scenario, TM emerged as the most effective and feasible method available to ensure continuous care, using smartphones apps and telephone or video calls [5]. Many interesting data from all over the world have been drawn from this experience, to support this method as a comparable system to the traditional visit for the management of the HF patient [5]. TM encompasses teleconsultation, telemonitoring, telerehabilitation (TR), shared electronic patient records, and medical teleconferencing [6]. The impressive technological development of the last decades led to a hypothetical role of avatar technology in treating HF patients by TM [7]. Furthermore, the use of artificial intelligence (AI) could help in managing the immense amount of data generated by TM [6].

As expected, the recent spread of TM has not been homogeneous worldwide due to differences between countries in terms of economy, health education, and availability of infrastructures [8]. Globally, two extremes of the diffusion of TM can be identified respectively in North-Middle Africa, where there are several limitations such as poverty and lack of infrastructure, and in the USA, where televisits have increased by more than 3000% in response to the COVID-19 pandemic. However, even in the USA, the TM employment still needs to be further regulated in order to be more accessible [8]. Even in Europe, there were several differences across countries: in Italy, for instance, TM has gained more and more importance after the pandemic and practical guidelines have been published with the aim of systematizing its role, but the limited information and communications technology infrastructures available in the country are still a limit for the TM use [8]. On the other hand, Switzerland had an excellent telemedical system already before the pandemic [9] ruled by specific guidelines [8].

After this experience, TM has confirmed to be an effective alternative for managing chronic diseases especially those with significant weight on the cost of healthcare [10]. Notably, HF is a chronic disease characterized by high mortality, morbidity, and the need for periodical visits. Low therapy compliance contributes to worsening of symptoms, in many cases, leading to hospitalization causing both a worsening of the clinical outcomes and a considerable financial burden upon healthcare systems [11]. Indeed nearly 13.9 million people suffer from HF worldwide and hospitalization accounts for almost 70% of total cost [12].

The purpose of this review is therefore to analyze the benefits that TM, correctly applied to selected HF patients, can provide in terms of early diagnosis, therapeutic management, and prognosis improvement.

## Telemedicine and Heart Failure

HF is defined as a multisystemic disease characterized by repeated hospitalizations and progressive worsening. HF patients are extremely prone to complications, and it is essential to provide a strict follow up with an extremely precise timing, particularly during the vulnerable phases between hospitalizations. A correct schedule of the follow-up visits remains a pivotal target to improve patients’ prognosis [5].

Especially after the COVID-19 outbreak, the potential of TM was highlighted at various points in the management of HF patients.

### Telemedicine and Heart Failure’s Primary and Secondary Prevention

In developed countries, coronary heart disease and hypertension are the two predominant etiologies of HF [2]. TM has shown to be efficient in blood pressure (BP) monitoring and in the prevention of the main cardiovascular risk factors (i.e., dyslipidemia, diabetes, smoking habit, and obesity) [13, 14•]. Even if hypertension is considered to be the most common cardiovascular risk factor, it is often unrecognized, and, in the long term, it can be responsible for a high rate of cardiovascular events. Home BP measurement is gaining importance, and the control of hypertension throughout the use of validated, automated BP devices makes possible the assessment of BP levels in different settings, time periods, seasons, and positions avoiding the “white coat effect” [13]. In the future, the ability to measuring BP with wearable devices could improve the self BP monitoring leading to a faster control in case of BP values persistently out of range [15].

Diabetes is another major risk factor associated with HF in developed countries [16]. Digital Diabetes Prevention Programs demonstrated effectiveness in improving weight, HbA1c, and lifestyle among people with prediabetes [14•]. Patients could access several tools such as maintaining contact with a trained lifestyle coach and monitoring physical activity [14•].

TM has also showed to be useful for the secondary prevention, helping patients who experienced a coronary heart event, to prevent mortality and recurrent events. For instance, Tobacco, Exercise and Diet Messages (TEXTME) study is aimed at examining the effect of a lifestyle support program for patients with coronary artery disease (CAD), based on text messaging. In this study, a significant reduction of low-density lipoproteins cholesterol (LDL-C) level was reached [17•].

Actually, the principal goal for chronic HF management is the early detection and diagnosis of a likely HF flare-up before it becomes clinically manifest, thereby reducing the need for hospitalization. It is essential for HF patients to avoid hospitalizations because of the high risk of nosocomial infections, hospital complications and immobilization, as well as for the reduction of healthcare costs. In a Consensus Document of the Italian Association of Hospital Cardiologists (A.N.M.C.O.), the Italian Society of Cardiology (S.I.C.), and the Italian Society for Telemedicine and eHealth (Digital S.I.T.), it has been observed that the remote control of vital signs can strongly prevent patients’ deterioration due to hypertension, anemia, infection, and renal failure [18].

Bashi et al. demonstrated that the remote patient monitoring (RPM) reduced HF rehospitalization and mortality by collecting vital signs such as BP, heart rate (HR), and electrocardiogram (ECG) [19]. The Telemedical Interventional Management in Heart Failure II (TIM-HF2) trial evaluated the benefit of RPM to detect early signs and symptoms of cardiac decompensation in order to initiate an appropriate treatment and care. The structured RPM was based on daily transmission of body weight (BW), systolic and diastolic BP, HR, heart rhythm’s analysis, peripheral capillary oxygen saturation, self-rated health status to the telemedical center, and periodic transmission of biomarker data. TIM-HF2 showed a reduction of the time spent in hospital for unplanned cardiovascular reasons and a reduction of all-cause mortality by using RPM [20]. Regarding BW monitoring, the “Innovative telemonitoring enhanced care program for chronic heart failure (ITEC-CHF)” study [21] is aimed at evaluating a possible increased compliance in BW monitoring through the use of ITEC-CHF program. This study showed the achievement of frequent BW monitoring and an improvement in self-care management.

During COVID-19 pandemics, cardiology centers were encouraged to use TM in order to monitor stable HF patients. In particular, in HF patients with cardiac implantable electronic devices (CIEDs), a lot of data like detection of arrhythmias and HR could be transmitted to clinicians. These devices were able to transmit data regarding intrathoracic impedance and early signs of HF allowing the medical team to undertake a timely clinical action [22]. In patients with CIEDs, information obtained by the devices has allowed a satisfying remote monitoring, reducing the infectious risk while maintaining high quality care [23•]. Remote monitoring can be rapidly activated and adopted with a comprehensive patient education plan [24].

Another mean to optimally control patients’ hemodynamic status may be the surveillance of the pulmonary artery pressure by implantable hemodynamic monitoring systems, although discordant results were highlighted so far. Among the main randomized trials, the “CardioMEMS Heart Sensor Allows Monitoring of Pressure to Improve Outcomes in New York Heart Association (NYHA) Class III Heart Failure Patients (CHAMPION)” trial showed that a pulmonary artery pressure guided management led to a significant reduction in admissions to hospital [25•]. The same results were obtained by the MONITOR-HF trial [26], which investigated the role of remote hemodynamic monitoring in improving quality of life and reducing HF hospitalization if added to standard care. Differently from the CHAMPION trial, in the MONITOR-HF trial, sodium-glucose co-transporter 2 inhibitors (SGLT2i) and angiotensin receptor/neprilysin inhibitor (ARNI) were part of the guideline-directed medical treatment (GDMT) leading to a much higher level of achievement of the GMDT [26]. In the future, through the use of algorithms based on ECG data, TM could also be decisive in intercepting worsening HF. AI could also be useful in organizing hospital, intensive care, and staffing needs, and it may be employed to interpret and understand a constantly increasing volume of data. However, the clinical relevance of the algorithms on which AI is based is not yet established because of the lack of their validation. In order to develop and validate AI programs, access to a vast amount of data is needed [27]. However, to restrict the risk of dehumanization, telehealth should be considered as an adjunct to face-to-face contact [28].

The fluid volume and congestion of the HF patients may be assessed by a rapid echocardiographic evaluation through the quantification of the caliber and collapse of the inferior vena cava. Echocardiography is recognized by the European society of cardiology (ESC) to play a key role in the HF diagnosis and management [2]. The use of tele-echocardiography has been proposed to reduce the work-load and overcome geographic challenges. Hjorth-Hansen et al. demonstrated that tele-echocardiography performed by specialized nurses associated with a remote interpretation by cardiologists has shown to provide reliable results and accurate [29]. Similarly, the use of hand-held ultrasound devices by general practitioners, combined with telemedical support by an external cardiologist, has shown to improve diagnostic precision in ruling in and ruling out HF [30].

In order to avoid hospital admissions as much as possible, a “far-from-hospital” management may be pivotal in the management of HF patients. An important strategy that can help to avoid hospitalizations, in addition to the administration of intra-venous (IV) loop diuretics in hospital settings, is the “home-hospitalization.” The first one consists of 3 h-IV diuretic intra-hospital infusion, depending on the maintenance diuretic dose; the “home-hospitalization” instead consists in home IV diuretic administration, and it strongly depends on the geriatric and social evaluation of the patient and its caregiver responsibility. These two manners of handling the IV loop diuretic therapy could be useful to improve quality of life and congestion of these “frequent flyers” patients [31].

However, results from different studies are discordant about primary and secondary prevention.

Buss et al. showed that a mobile health-based interventions had a scarce effectiveness in controlling cardiovascular disease risk factors and diabetes mellitus type 2 [32]. These contrasting experiences require further investigations. Furthermore, Bae et al. aimed to evaluate the effect of short message service (SMS)-text messages in the improvement of lifestyle modifications, and medication adherence. However, the impact on LDL-C levels, systolic BP, and BMI was not significant [33]. Other lacking evidence regards the actual advantages of TM in preventing HF hospitalizations: Chaundhry et al.’s study, which followed up patients who had recently been hospitalized for HF with two different methods: the telemonitoring group did not show a decrease of hospitalizations if compared to the “standard of care” arm [34]. For what concerns the invasive hemodynamic-based management of HF patients, the GUIDE-HF trial showed that the management of HF patients using an implantable pulmonary artery pressure monitor did not lead to a lower mortality or total HF events; however, it is interesting to notice that a pre-COVID-19 impact analysis demonstrated a significant decrease in HF hospitalizations [35].

### Optimization of the Guideline-Directed Medical Treatment

The 2021 ESC Guidelines for the diagnosis and treatment of acute and chronic HF recommend a management of the chronic HF with reduced ejection fraction (HFrEF) based on the administration of angiotensin-converting enzyme inhibitors (ACE-I) or ARNI, beta-blockers (BB), mineralocorticoid receptor antagonists (MRA), and SGLT2i [2]. Despite the recommendations to achieve GMDT, only a small percentage of patients are able to reach the target dose, and this goal was further complicated by COVID-19 outbreak. In addition, more than a half of HF patients have comorbidities linked to the impossibility to achieve the GMDT, as for the concomitant presence of kidney failure that does not allow full intake of HF drugs [34, 36]. Moreover, it is known that an optimized medical therapy should be reached as soon as possible after an index hospitalization due to HF, in order to reduce mortality and the risk of a new hospitalization. This strategy can be applied only if patients are frequently evaluated after discharge. However, it is difficult to guarantee so frequent visits in the HF-dedicated outpatient service. Thus, in this context, TM fits with all its advantages. In fact, to fully up-titrate the GMDT, a clinical assessment is necessary, and it has been shown to be feasible by using TM [37•]. Several recent studies have demonstrated the usefulness of TM in obtaining GMDT, both during hospitalization and the follow-up. Rao et al. performed in-hospital virtual peer-to-peer consultation. The goal was to achieve a GMDT thanks to a communication between a HF-team and rounding medical teams to correctly assess patients’ eligibility to GMDT [38]. Virtual visits have been shown to achieve fewer discontinuation of GMDT compared to the usual care. These results are concordant with the IMPLEMENT-HF trial that aimed to assess the achievement of GMDT through an entirely virtual platform with no direct communication with patients [39, 40]. The use of the virtual platform resulted in a proper optimization of GMDT which was also continued in the post-discharge follow-up [39].

Moreover, in a prospective implementation trial, a virtual care team, tele-guided strategy improved GMDT, doubling BB prescription, and tripling MRA prescription without increasing the hospital length of stay [40].

For example, the adjustment on diuretic dose is based on the volume assessment and can be easily evaluated by the patient himself, if well instructed on how to control daily BW, jugular venous distension, peripheral edema, orthopnea, and New York Heart Association (NYHA) Class. Furthermore, electrolytes, renal function, but also serum HF markers as brain natriuretic peptide (BNP) can be examined by asking laboratory essays during virtual visits [37•]. BP and HR monitoring, even telematic, could be useful for up-titrate BB, ACE-I, or ARNI therapy.

The achievement of the GMDT in HF patients is also complicated by concomitant polytherapy. Polypharmacy is defined as the need of taking more than 5 medications daily, and it can represent an overwhelming routine for the patient. In this scenario, telemonitoring HF patients has shown to significantly increase treatment adherence leading to decreased rehospitalizations, lowering health-related costs, and reduction in mortality [36]. It is also important to consider the inclusion of non-physician personnel guided by a HF specialist to supplement patients’ clinical follow up [41].

Medication non-adherence has been identified by the World Health Organization (WHO) as a preventable healthcare problem. Gandapur et al. investigated the possible role of mobile health in improving medication adherence [42]. The most used type of telehealth was the text messaging in combination with other interventions. Anyway, a significant improvement in medication adherence was reported in all the studies. Adherence in older patients could be improved by TM medication reminders, even if the current results are not totally consistent with one another. Goldstein et al., for example, proposed a telehealth strategy based on the use of alarmed electronic pillbox or a smartphone to increase patient adherence to their polytherapy, and even if they both demonstrated a high overall adherence rate, probably due to the characteristics of the sample, they failed to prove a significant global change of therapy adherence [43].

### The Role of Telemedicine on HF Prognosis

The natural history of HF patients is marked by continuous exacerbations, subsequent hospitalizations, and progressive impoverishment of the prognosis. However, the HF patients’ prognosis strongly depends on a precise clinical handling, constant optimization of therapy, strict control of risk factors, and high attention to any change in symptoms and/or clinical signs and/or laboratory tests. This delicate and continuous link between patients and physicians is often difficult to uphold, especially in big hospitals’ realities, where the number of patients is very high or when patients are elderly and fragile and can have difficulties in constantly presenting for regular visits. The TM can overcome all these limitations. The risk of readmission after hospital discharge appears to be high in HF patients requiring an active outpatient intervention. According to recent data on HF hospitalizations, this intervention could detect more than a half of the causes of decompensation potentially avoiding the hospitalization [44]. Liprandi et al. demonstrated that the implementation of non-invasive TM follow-up showed a high degree of clinical stability and a low rate of events on 12 months of follow-up [44]. Especially during COVID-19 pandemic, TM decreased the risk of infection, allowing patients to have an easier access to care [36], but the advantages of TM for HF frail patients, as listed above, are evident even outside the COVID-19 era. The use of TM in HF patients’ management can include various kind of intervention. Teleconsultation has shown to be a valid option to ensure continuous care allowing healthcare providers to constantly evaluate the patient’s health status, avoiding the disease exacerbation facing it before the need of hospitalization. It has also been proved as a valid option to facilitate patients who could not attend an HF outpatient in-person visit reducing the caregivers’ burden [5].

In addition, self-management appears to have an important role in HF patients’ prognosis. Self-care can be defined as the set of behaviors that aims to avoid HF worsening and to identify and control signs and symptoms. This important issue has been examined in MOTIVATE‐HF trial [45] which evaluated the effects of motivational interviewing in the improvement of self-care maintenance and changes in self-care over time. Motivational interview has been shown to be an important and inexpensive tool that can improve outcomes.

The effect that TM can have in terms of mortality in chronic HF patients has been examined by the TIM-HF trial [46]. In this trial, a wireless Bluetooth system with a personal digital assistant was used in the intervention group. ECG, BP, and BW measurements were transmitted to the telemedical centers. The primary outcome of all-cause mortality was not lower in the intervention group if compared to usual care, in particular, if applied to stable and optimally treated patients [46]. The same results were highlighted in TELE-HF trial, showing an absence of reduction in the risk of HF hospitalization or all-cause mortality in the TM group [34]. The patients enrolled in this trial had been hospitalized for worsening HF and the TM program consisted of daily calls presenting to the patient questions about general health and HF symptoms [34].

Lastly, HFrEF entity represents the most studied HF group, for which the pathophysiology as well as the therapeutic options is well established. On the other hand, the pathophysiology and the treatment available for HF with mildly reduced ejection fraction (HFmrEF) and HF with preserved ejection fraction (HFpEF) patients remain unclear. In these populations, TM may play an additive role, being a tool to prevent hospitalizations, thus improving the prognosis [47, 48]. A subanalysis of the recent ICOR study aimed to evaluate the efficacy of TM in these patients, in terms of risk of non-fatal acute HF and risk of hospitalization after 6 months of follow-up [49]. This sub analysis highlighted a reduced risk of the two primary outcomes if compared with usual care. In particular, the risk of HF hospitalizations could be reduced by timely treatment adjustments [49].

As for the HF prognosis, fundamental dilemmas on the use of this tool are still open. Many questions concern what type of patients, how, and for how long should be followed up with TM, while other unsolved issues concern who should run the tele-visits and who should intervene in emergency case [50].

Also, the AHA/ACC/HFSA (American College of Cardiology/American Heart Association Science/Heart Failure Society of America) guidelines declare that further studies regarding the use of TM are necessary to set up before its systematic introduction into clinical practice [51].

Some authors speculate that the effectiveness of telemonitoring varies according to the frequency of hospital HF treatment and mortality and that in more stable patients, it has not yet been definitively clarified. In fact, some divergent evidence may be explained by considering the differences in telemonitoring methodology and the target population characteristics [52].

## Conclusion

The continuous increase of the amount of HF patients and the recent experience of the COVID-19 pandemic proved that new health management strategies need to be involved in order to optimize the available resources. Although it has not yet fully entered in the daily clinical practice, different studies have shown that TM can be a turning point for HF management by different points of view, as summarized in Table 1. TM has proven to be effective both in the primary and in the secondary prevention. TM could also help to achieve GMDT, currently one of the most debated and challenging goals in the HF field, consequently reducing hospitalizations and mortality and improving the quality of life and lowering the economic pressure on health systems.

**Table 1:** Main studies highlighting the role of TM in the different settings of HF management

TM and HF management setting	Main evidence	References
Primary prevention	- TM has shown to be efficient in controlling BP	[13]
	- Digital Diabetes Prevention Programs demonstrated effectiveness in improving weight, HbA1c, and lifestyle among people with prediabetes	[14•]
	- Mobile apps and SMS text messages may encourage the changing of multiple behavioral risk factor	[18]
Secondary prevention	- RPM reduced HF rehospitalization and mortality by collecting vital signs such as BP, heart rate, and electrocardiogram; it also reduced the time spent in hospital	[21, 23•]
	- Pulmonary artery pressure guided management may lead to a significant reduction in admissions to hospital	[28–30]
	- CIEDs are able to transmit data regarding intrathoracic impedance, and early signs of HF allowing the medical team to undertake a timely clinical action	[26]
GDMT optimization	- TM has shown to be effective in obtaining an adequate uptitration of HF therapy, improving also therapeutic adherence	[37•, 39–41, 43]
Prognosis	- TM decreased the risk of infection, allowing patients to have an easier access to care - Teleconsultation has shown to be a valid option to ensure continuous care allowing healthcare providers to constantly evaluate the patient’s health status, avoiding the disease exacerbation facing it before the need of hospitalization	[21, 45, 46, 49]

*TM*, telemedicine; *HF*, heart failure; *BP*, blood pressure; *HbA1c*, hemoglobin A1C; *SMS*, short message service; *RPM*, remote patient monitoring; *CIEDs*, cardiac implantable electronic devices; *GDMT*, guideline directed medical therapy

Although current studies have shown promising results, some of them have shown mixed results. Moreover, there are still gaps in terms of the appropriate duration of TM programs, the selection of patients that can benefit more or less from this system, and finally, what relationship should exist between TM and traditional in-person visits [53]. Further efforts will be needed to achieve unambiguous results on the usefulness of this tool in HF patients’ management.

## References

[1] Conrath DW (1975). An experimental evaluation of alternative communication systems as used for medical diagnosis. Behav Sci.

[2] McDonagh TA (2021). 2021 ESC Guidelines for the diagnosis and treatment of acute and chronic heart failure: developed by the Task Force for the Diagnosis and Treatment of Acute and Chronic Heart Failure of the European Society of Cardiology (ESC) with the special contribution of the Heart Failure Association (HFA) of the ESC. Eur Heart J.

[3] Wootton R, Trafton JA, Gordon WP (2003). Chronic disease management telemedicine. pp 1–20 in Best practices in the behavioral management of chronic disease. Vol II Other medical disorders.

[4] Wootton R (2012). Twenty years of telemedicine in chronic disease management–an evidence synthesis. J Telemed Telecare.

[5] Severino P (2022). Clinical support through telemedicine in heart failure outpatients during the COVID-19 pandemic period: results of a 12-months follow up. J Clin Med.

[6] Silva-Cardoso J, et al. The future of telemedicine in the management of heart failure patients. Cardiac Fail Rev. 2021:7. 10.15420/cfr.2020.32. 10.15420/cfr.2020.32 PMC8201465 34136277

[7] Gingele AJ, et al. Integrating avatar technology into a telemedicine application in heart failure patients: a pilot study. Wiener klinischeWochenschrift. 2023: 1–5. 10.1007/s00508-022-02150-8.

[8] Omboni S (2022). The worldwide impact of telemedicine during COVID-19: current evidence and recommendations for the future. Connect Health.

[9] Nittas V, von Wyl V (2020). COVID-19 and telehealth: a window of opportunity and its challenges. Swiss Med Wkly.

[10] Waters HUGH, Marlon G (2018). The costs of chronic disease in the US.

[11] Vermeire E, Hearnshaw H, Van Royen P (2001). Patient adherence to treatment: three decades of research. A comprehensive review. J Clin Pharm Ther.

[12] van Der Wal MHL (2006). Compliance in heart failure patients: the importance of knowledge and beliefs. Eur Heart J.

[13] Citoni B (2022). Home blood pressure and telemedicine: a modern approach for managing hypertension during and after COVID-19 pandemic. High Blood Pressure Cardiovasc Prev.

[14] • Katula JA, et al. Effects of a digital diabetes prevention program: an RCT. Am J Prev Med. 2022; 62(4): 567–577. 10.1016/j.amepre.2021.10.023. **Digital Diabetes Prevention Programs demonstrated promising results in improving the lifestyle, notably the weight and HbA1c among people with prediabetes.** 10.1016/j.amepre.2021.10.023 35151522

[15] Ruberti OM (2021). Hypertension telemonitoring and home-based physical training programs. Blood Press.

[16] Kannel WB, McGee DL (1979). Diabetes and cardiovascular risk factors: the Framingham study. Circulation.

[17] • Chow CK, Redfern J, Hillis GS, Thakkar J, Santo K, Hackett ML, et al.. Effect of lifestyle-focused text messaging on risk factor modification in patients with coronary heart disease: a randomized clinical trial. JAMA. 2015; 314(12):1255–1263. 10.1001/jama.2015.10945.2442937. **Mobile apps and SMS text messages may encourage the changing of multiple behavioral risk factor, in particular of LDL cholesterol.** 10.1001/jama.2015.10945 26393848

[18] Di Lenarda A (2017). The future of telemedicine for the management of heart failure patients: a Consensus Document of the Italian Association of Hospital Cardiologists (ANMCO), the Italian Society of Cardiology (SIC) and the Italian Society for Telemedicine and eHealth (Digital SIT). Eur Heart J Suppl.

[19] Bashi N (2017). Remote monitoring of patients with heart failure: an overview of systematic reviews. J Med Internet Res.

[20] Koehler F (2018). Efficacy of telemedical interventional management in patients with heart failure (TIM-HF2): a randomised, controlled, parallel-group, unmasked trial. Lancet.

[21] Ding H (2020). The effects of telemonitoring on patient compliance with self-management recommendations and outcomes of the innovative telemonitoring enhanced care program for chronic heart failure: randomized controlled trial. J Med Internet Res.

[22] Imberti JF (2021). Remote monitoring and telemedicine in heart failure: implementation and benefits. Curr Cardiol Rep.

[23] • Piro A, Magnocavallo M, Della Rocca DG, Neccia M, Manzi G, Mariani MV, et al. Management of cardiac implantable electronic device follow-up in COVID-19 pandemic: lessons learned during Italian lockdown. J Cardiovasc Electrophysiol. 2020;31: 2814–2823. **CIEDs are able to transmit data regarding intrathoracic impedance and early signs of HF allowing the medical team to undertake a timely clinical action.** 10.1111/jce.14755 PMC7646650 32954600

[24] Magnocavallo M, Bernardini A, Mariani MV, Piro A, Marini M, Nicosia A, Adduci C, Rapacciuolo A, Saporito D, Grossi S (2021). Home delivery of the communicator for remote monitoring of cardiac implantable devices: a multicenter experience during the COVID-19 lockdown. Pacing Clin Electrophysiol.

[25] • Abraham WT, et al. Sustained efficacy of pulmonary artery pressure to guide adjustment of chronic heart failure therapy: complete follow-up results from the CHAMPION randomised trial. Lancet. 2016; 387(10017): 453–461. **Pulmonary artery pressure guided management may lead to a significant reduction in admissions to hospital.** 10.1016/S0140-6736(15)00723-0 26560249

[26] Brugts JJ, et al. Remote haemodynamic monitoring of pulmonary artery pressures in patients with chronic heart failure (MONITOR-HF): a randomised clinical trial. Lancet (2023). 10.1016/S0140-6736(23)00923-6 37220768

[27] Leclercq C, et al. Wearables, telemedicine, and artificial intelligence in arrhythmias and heart failure: Proceedings of the European Society of Cardiology Cardiovascular Round Table. Europace. 2022; 24(9): 1372–1383. 10.1093/europace/euac052 35640917

[28] D'Amario D, Canonico F, Rodolico D, Borovac JA, Vergallo R, Montone RA, Galli M, Migliaro S, Restivo A, Massetti M, Crea F (2020). Telemedicine, artificial intelligence and humanisation of clinical pathways in heart failure management: back to the future and beyond. Card Fail Rev.

[29] Hjorth-Hansen AK (2020). Feasibility and accuracy of tele-echocardiography, with examinations by nurses and interpretation by an expert via telemedicine, in an outpatient heart failure clinic. J Ultrasound Med.

[30] Magelssen MI (2022). The clinical influence of hand-held ultrasound examinations by general practitioners in patients with suspected heart failure supported by tools for automatic quantification and telemedicine. Eur Heart J-Cardiovasc Imaging.

[31] Girerd N (2022). Practical outpatient management of worsening chronic heart failure. Eur J Heart Fail.

[32] Buss VH (2020). Primary prevention of cardiovascular disease and type 2 diabetes mellitus using mobile health technology: systematic review of the literature. J Med Internet Res.

[33] Bae JW (2021). mHealth interventions for lifestyle and risk factor modification in coronary heart disease: randomized controlled trial. JMIR Mhealth Uhealth.

[34] Chaudhry SI, Mattera JA, Curtis JP, Spertus JA, Herrin J, Lin Z (2010). Telemonitoring in patients with heart failure. N Engl J Med.

[35] Lindenfeld J, Zile MR, Desai AS, Bhatt K, Ducharme A, Horstmanshof D (2021). Haemodynamic-guided management of heart failure (GUIDE-HF): a randomised controlled trial. Lancet.

[36] Hernandez LS (2022). Role of telemedicine in improving guideline-directed medical treatment for patients with heart failure during a pandemic. Crit Care Nurs Clin N Am.

[37] • Thibodeau JT, and Gorodeski EZ. Telehealth for uptitration of guideline-directed medical therapy in heart failure. Circulation. 2020; 142(16): 1507–1509. **TM has shown to be effective in obtaining an adequate uptitration of HF therapy, improving also therapeutic adherence.** 10.1161/CIRCULATIONAHA.120.050582 33074759

[38] Rao VN (2023). In-hospital virtual peer-to-peer consultation to increase guideline-directed medical therapy for heart failure: a pilot randomized trial. Circ Heart Fail.

[39] Bhatt AS (2021). Virtual optimization of guideline-directed medical therapy in hospitalized patients with heart failure with reduced ejection fraction: the IMPLEMENT-HF pilot study. Eur J Heart Fail.

[40] Bhatt AS (2023). Virtual care team guided management of patients with heart failure during hospitalization. J Am Coll Cardiol.

[41] Desai AS (2020). Remote optimization of guideline-directed medical therapy in patients with heart failure with reduced ejection fraction. JAMA Cardiol.

[42] Gandapur Y (2016). The role of mHealth for improving medication adherence in patients with cardiovascular disease: a systematic review. Eur Heart J-Qual Care Clin Outcomes.

[43] Goldstein CM (2014). Randomized controlled feasibility trial of two telemedicine medication reminder systems for older adults with heart failure. J Telemed Telecare.

[44] Sosa Liprandi MI, Elfman M, Zaidel EJ, Viniegra M, Sosa Liprandi Á. Impact of a telemedicine program after a heart failure hospitalization on 12 months follow-up events. Curr Probl Cardiol. 2023;48(6):101624. 10.1016/j.cpcardiol.2023.101624. 10.1016/j.cpcardiol.2023.101624 36724818

[45] Vellone E (2020). Motivational interviewing to improve self-care in heart failure patients (MOTIVATE-HF): a randomized controlled trial. ESC Heart Fail.

[46] Koehler F (2011). Impact of remote telemedical management on mortality and hospitalizations in ambulatory patients with chronic heart failure: the telemedical interventional monitoring in heart failure study. Circulation.

[47] Severino P (2022). Do the current guidelines for heart failure diagnosis and treatment fit with clinical complexity?. J Clin Med.

[48] Severino P (2023). Heart failure pharmacological management: gaps and current perspectives. J Clin Med.

[49] Jiménez-Marrero S (2020). Impact of telemedicine on the clinical outcomes and healthcare costs of patients with chronic heart failure and mid-range or preserved ejection fraction managed in a multidisciplinary chronic heart failure programme: a sub-analysis of the iCOR randomized trial. J Telemed Telecare.

[50] Krzesiński P (2023). Digital health technologies for post-discharge care after heart failure hospitalisation to relieve symptoms and improve clinical outcomes. J Clin Med.

[51] Writing Committee Members; ACC/AHA Joint Committee Members (2022). 2022 AHA/ACC/HFSA Guideline for the Management of Heart Failure. J Card Fail.

[52] Stockburger M (2017). Non-device-based telemonitoring: toy or tool?. Herzschrittmacherther Elektrophysiol.

[53] Zhu Y, Xiang G, Chao X (2020). Effectiveness of telemedicine systems for adults with heart failure: a meta-analysis of randomized controlled trials. Heart Fail Rev.

